# HUMANNET—A Two-Tiered Deep Neural Network Architecture for Self-Occluding Humanoid Pose Reconstruction

**DOI:** 10.3390/s21123945

**Published:** 2021-06-08

**Authors:** Audrius Kulikajevas, Rytis Maskeliunas, Robertas Damasevicius, Rafal Scherer

**Affiliations:** 1 Department of Multimedia Engineering, Kaunas University of Technology, 51368 Kaunas, Lithuania; audrius.kulikajevas@ktu.lt (A.K.); rytis.maskeliunas@ktu.lt (R.M.); 2 Faculty of Applied Mathematics, Silesian University of Technology, 44-100 Gliwice, Poland; 3 Department of Intelligent Computer Systems, Częstochowa University of Technology, 42-200 Częstochowa, Poland; rafal.scherer@pcz.pl

**Keywords:** 3D shape recognition, 3D depth scanning, pointcloud reconstruction, human shape reconstruction

## Abstract

Majority of current research focuses on a single static object reconstruction from a given pointcloud. However, the existing approaches are not applicable to real world applications such as dynamic and morphing scene reconstruction. To solve this, we propose a novel two-tiered deep neural network architecture, which is capable of reconstructing self-obstructed human-like morphing shapes from a depth frame in conjunction with cameras intrinsic parameters. The tests were performed using on custom dataset generated using a combination of AMASS and MoVi datasets. The proposed network achieved Jaccards’ Index of 0.7907 for the first tier, which is used to extract region of interest from the point cloud. The second tier of the network has achieved Earth Mover’s distance of 0.0256 and Chamfer distance of 0.276, indicating good experimental results. Further, subjective reconstruction results inspection shows strong predictive capabilities of the network, with the solution being able to reconstruct limb positions from very few object details.

## 1. Introduction

Computer vision is a quickly expanding field because of the success of deep neural networks [1]. The RGB camera frames have already been adopted in various industries for environment recognition [2] and object detection [3] tasks. Depth information is however, is less likely to be used due to generally requiring special sensors or monocular camera setups. For this reason computer vision field has a lot of open questions regarding the application of depth information. One of important computer vision research fields, related to application of depth information, is three-dimensional object reconstruction [4].

A lot of applications that would benefit from real-time object reconstruction such as self-driving cars [5,6], interactive medium particularly virtual reality [7] (VR) and video games, augmented reality [8] (AR) and extended reality [9] (XR). Furthermore, depth sensor information can improve gesture [10,11] and posture recognition [12] technologies as these tasks generally have a lot of important depth information embedded into them. Additional uses for object reconstruction from depth sensor information could include recreating environments in film industry and teleconferencing with the use of holograms, indoor mapping [13] or robotics [14,15]. Unfortunately, while this object reconstruction gives a lot of value to various fields, generally such applications require intricate camera setups to scan the entire object from all sides or to move camera in order to gradually build the object depth profile. This makes the reconstruction technology have a high barrier of entry.

Users cannot be forced to have professional filming setups containing laser sensor arrays that would scan entire object from all perspectives in a single shot, or expect user to bother scanning the object from all sides to reconstruct it each time they add additionally obstacles to the scene. In addition, it potentially requires a lot of technical know-how and computing power to perform high fidelity pointcloud fusion, this reduces the end-user experience. For this reason, there is a need for different type solution which is capable of performing such task using only a single view. Some novel state-of-the-art methods already attempt to solve this problem using a priori knowledge. Such methods generally involve using black-box models such as deep neural networks as it gives the approaches ability to approximate the occluded object information that is generally quite easy for a person to infer based on the mental model each of us builds over our lifespans. Initial successful research in the object reconstruction field has focused in the voxel based reconstruction [16]. The proposed approach dubbed 3D-R2N2 has used Sanford Online Products [17] and ShapeNet [18] datasets as a priori knowledge to guess object shape using multi-view reconstruction. Other research has improved the results with the addition of Chamfer Distance as a loss function [19] thus increasing the reconstruction accuracy. Other attempts have attempted improving the reconstruction by using network hybridization where each network branch is trained on different group of objects thus allowing for faster model convergence and real-time reconstruction [20]. While all the mentioned methods focus on single object per scene reconstruction, there have been attempts in improving this with the use of object segmentation layer [21]. By segmenting only necessary depth information and using that as reconstruction it allows for multiple object per scene.

While the majority of methods focus on voxel based mesh representation [22,23,24,25,26,27], for object reconstruction due to their representation simplicity, voxels have one major flaw—exponentially increasing requirements to train them with increasing fidelity. Some papers tried to solve this ever-increasing memory requirements using smarter data representation styles like octrees [28,29]. These allow for more details to be preserved, however, they still are not as detailed as pointclouds. There already exists some solutions that attempt to do this such as PointOutNet [30] that has shown the ability to predict and generate plausible 3D shapes of objects. While this solution has shown generally good prediction results, it relies on user segmentation mask for reconstruction. While PointOutNet is capable of leveraging 2D convolutions in order to reconstruct 3D object, there is some information that is missing for this approach to be stable. Even though 3D convolutions can be easily applied to voxel clouds both 2D and 3D convolutions are not very useful when dealing with pointclouds as they have fundamentally different structure. Some approaches configurations have shown the ability to generalize pointcloud information [31]. Further modifications to PointNet have been shown to be able to reconstruct shapes using pointcloud inputs [32].

We propose a novel two tiered approach capable of full human body pointcloud reconstruction using a single realistic imperfect (self-occluding) depth view, where the first rank network clips the initial depth cloud and the second rank uses prime output to reconstruct the captured object. Our contribution to the field of object reconstruction is the addition of the clipping-resampling node which gives our approach the ability to extract three-dimensional Regions of Interest (RoIs) that can be then used for reconstruction. Unlike previous existing approaches which rely on user-defined masks to extract regions of interest, ours is completely independent and provides a complete solution sensor-to-screen object reconstruction.

Generally, reconstruction focuses on static single object per scene reconstruction. However, we attempt to reach new a frontier in this field. Our approach attempts to take one step further, reconstruction of full human shape using single imperfect depth frame information in order to reconstruct missing scene information. Our method involves two tiered reconstruction networks and a priori knowledge of the human body to make the predictions of the reconstructed pose.

## 2. Related Work

Object reconstruction is a rapidly expanding computer vision field. Most of the new solutions that relate to this topic benefit from the advancements in the artificial intelligence. Two main approaches for three-dimensional object reconstruction are: voxel based and pointcloud based. One such voxel based solution is 3D-R2N2. It uses Long Short Term Memory [33,34] (LSTM) in order to learn the object features from multiple views and later reconstruct them. This approach is afterwards capable of reconstructing voxel grid using only a single RGB view based on a priori knowledge obtained during training. The method requires additional masks provided separately in order to reconstruct the results. Another solution attempted to use an extended YoloV3 [21] (YoloExt) has attempted to get rid of this dependency by merging YoloV3 [35] with the reconstruction task. Unlike prior solution the YoloExt was capable of detecting and then segmenting the RoIs itself and passing them mask and depth to the reconstruction branches. This allowed for the solution to be independent of additional user input and could work with real world data. However, the voxel based solutions while being simple to train suffer from two major flaws: exponential memory requirements to train and requiring high granularity grid in order to preserve small features. To resolve high memory requirements while maintaining high fidelity another competing reconstruction approach exists, i.e., pointcloud reconstruction. Unlike previous approaches it has a much lower memory impact, therefore potentially allowing for much higher fidelity reconstruction. However, the pointcloud solutions are notoriously hard to train due to a more complex loss function being required.

One of first such solutions was PointOutNet. Just like 3D-R2N2 it requires an external mask provided to the network and reconstructs the shape using RGB frames. However, unlike 3D-R2N2 it reconstructs the shape using unstructured pointcloud. Thus obtaining higher efficiency than the competing voxel approaches. The approach suggests both Chamfer and Earth Mover’s distance as loss metrics.

Further research in pointcloud reconstruction in PointNet [36] has attempted to instead of using RGB frame as input using a pointcloud. However, such pointcloud methods are unable to use the traditional 2D convolutions due to pointclouds being unstructured dataset. To solve for this problem, PointNet attempts to learn symmetric functions and learn local features. The addition of fully-connected auto-encoders to the PointNet has shown the ability to fill in missing chunks of the malformed pointcloud. PCN [37] proposes a fine-grained pointcloud completion method while maintaining a small number of training parameters due to its coarse-to-fine approach. AtlasNet [38] proposes a patches based approach capable of mapping 2D information into parametric 3D objects. Due to high complexity of $O(n^{2})$ required for the calculation of Earth Mover’s distance the majority of solutions tend to use Chamfer distance as loss metric. However, the latter is less sensitive to density distribution. For this reason, MSN [39] proposes an Earth Mover’s approximation which can be applied to pointclouds and a sampling algorithm for obtaining evenly distributed subset of pointcloud. However, all prior approaches all revolve around reconstructing quite static objects and not dynamically morphing meshes such as human body. Some approaches dealing with human body prediction using depth information exist [40,41,42,43] however their body predictions do not deal with full body reconstruction and only pose estimation.

The comparison of existing methods versus ours can be seen in Table 1, as we can see our solution is capable reconstructing sensor-to-screen pointclouds using only sensor provided information, while maintaining sensitivity to high density distributions due to the use of EMD as loss metric.

## 3. Materials and Methods

### 3.1. Proposed Deep Neural Network Architecture

**Figure 1:**
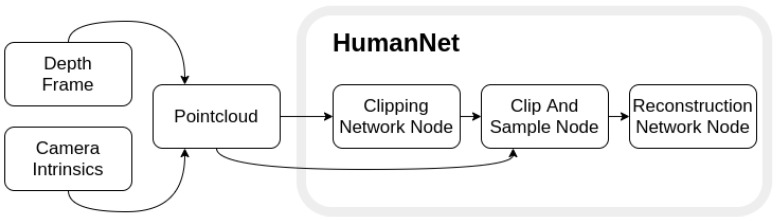
Proposed two-tiered network overview. Intrinsic camera matrix is applied to depth information in order to generate pointcloud. Pointcloud is then passed onto Clipping Network Node which finds predict the bounding box. The bounding box is then used along with initial point cloud to clip the Region of Interest and downsample. The result is then used to reconstruct the human shape.

Our synthetic dataset attempted to create real-world like dataset that other approaches were incapable of generalizing. For this reason our proposed black-box model (artificial neural network) consisted of two tier network structure (see Figure 1). The first network rank dealt with extracting the required features of the pointcloud and downsampling. The second rank uses the clipped and resampled pointcloud in order to learn the required features for full human body reconstruction.

### 3.2. Clipping Network Architecture

Our dataset involved two inputs: pinhole depth image and camera intrinsic matrix *K* (see Equation (1)). By applying camera intrinsics to each of depth points we created undistorted pointcloud that we could use for training. The first rank network (see Table 2) was responsible for filtering as much unnecessary information that the pointcloud contains as possible. This was done to avoid poisoning the initial neural network training states as they were tightly dependent on the input frame during training. Having too much unnecessary information made the reconstruction network very difficult to train. For this reason the main purpose of the first rank was to detect the desired feature bounding box.

One of the approaches to mask out only interesting data is to try and predict the 2D mask by using segmentation techniques capable of segmenting objects in the frame [44,45,46]. While such approaches can easily exploit 2D convolutions they lack one very important feature—a third dimension. Therefore, we would be unable to filter out objects that are in front of the object. Additionally, 2D convolutions are much slower than the approach we chose that dealt with pointclouds directly. Because our input depth resolution was 640 × 480 pixels once converted into pointcloud (see Equation (2)) we got a total of 307,200 vertices in the cloud. While it was possible to use this entire pointcloud as the neural network input it would make it unusable in real-time applications. For this reason we used Farthest Point Sampling [47] (FPS) operation to collect 2048 points. We found that this amount of vertices was more than enough to extract all necessary features from frames. The downsampled input was then used as an input for the network.

While the network was capable of learning most of the feature bounding boxes it was heavily biased by the imbalances of the dataset. Our dataset contained two primary types of bounding boxes tall-thin and short-wide due to two main human poses being either standing or crouching. For this reason we borrowed a widely used approach in Single Shot Detection methods where anchor boxes are used to help neural network learn the 2D object bounding boxes [48,49,50]. However, if we only had two anchor boxes our dataset would become very imbalanced, for that reason we increased the anchor count to four anchors, this gave us a more even pose distribution. The predicted three-dimensional bounding box acted as six clipping planes that allowed us to filter out all vertices that did not belong to that object.

(1) $$K=\left[\begin{array}{ccc} f_{x} & 0 & c_{x}\\ 0 & f_{y} & c_{y}\\ 0 & 0 & 1 \end{array}\right]$$

(2) $$p_{\left(x,y,z\right)}=\left[\begin{array}{c} \frac{z\cdot (c_{x}-x_{i})}{f_{x}}\\ \frac{z\cdot (c_{y}-y_{i})}{f_{y}}\\ z \end{array}\right]$$

Due to the fact that our approach had four potential bounding box anchors we got four potential bounding boxes. However, our network also outputted the confidence level of the bounding box. The bounding box with the highest confidence level was used for clipping. Once the highest confidence bounding box was acquired we could perform clipping and resampling operation using the initial 307200 vertex pointcloud. As our initial downsampling included points that did not belong to the Region of Interest the resulting point cloud had a much lower density, hence less information that could be used for reconstruction. For this reason we clipped the original pointcloud and downsampled to 4096 points. While it may seem counter-productive to resample twice instead of having the initial resampling with much higher density, however, FPS was a cheaper operation than working with a much higher pointcloud resolution.

(3) $$\epsilon_{clip}\left(y,\hat{y}\right)=\sum L1_{s}\left(y_{pos},{\hat{y}}_{pos}\right)\cdot y_{conf}+\sum L1_{s}\left(y_{scl},{\hat{y}}_{scl}\right)\cdot y_{conf}+\epsilon_{bce}\left(y_{conf},{\hat{y}}_{conf}\right)$$

When training our neural network we calculated three different loss functions: position loss, scale loss and confidence loss (see Equation (3)). $L1_{s}$ in Equation (3) refers to smooth L1 loss (see Equation (5)) [51], while BCE refers to binary cross entropy loss (see Equation (4)),

(4) $$\epsilon_{bce}\left(y,\hat{y}\right)=-\frac{1}{n}\cdot \sum_{i}^{n}y_{i}\cdot \log{\hat{y}}_{i}+\left(1-y_{i}\right)\cdot \log\left(1-{\hat{y}}_{i}\right)$$

(5) $$L1_{s}\left(y,\hat{y}\right)=\frac{1}{n}\sum_{i}^{n}z_{i}$$

where $z_{i}$ is Equation (6) with β=0.1.

(6) $$z_{i}\left(y_{i},{\hat{y}}_{i}\right)=\left\{\begin{array}{cc} \frac{0.5\cdot {\left({\hat{y}}_{i}-y_{i}\right)}^{2}}{\beta }, & if\;|{\hat{y}}_{i}-y_{i}|<\beta \\ |{\hat{y}}_{i}-y_{i}|-0.5\cdot \beta , & otherwise \end{array}\right.$$

### 3.3. Reconstruction Network Architecture

Our second rank network (see Table 3) was heavily inspired by Morphing and Sampling Network (MSN) which shows state-of-the-art reconstruction results for pointcloud reconstruction. However, the proposed network got easily poisoned by excess information that did not belong to the object which was being reconstructed, as it was heavily influenced by the initial pointcloud used as input.

As we can see from the Table 4, the modifications we made to the deep neural network architecture, had an overall negligible impact in terms of trainable parameters our neural network had to learn weights for and the model size, while slightly reducing the overall number of operations for the network to process due to the addition of resampling after clipping the objects RoIs.

Because the reconstruction network could easily get poisoned by bad input data due to its dependence on initial point positions, clipping loss had to reach ϵ<0.3 before reconstruction starts weights got updated. This approach kept randomized initial weight values in stable positions, easing the training process. The reconstruction training process requires a metric in order to compare ground truth *S* and prediction $\hat{S}$ values. While one of the most popular metrics when comparing pointclouds is Chamfer Distance [52] due to its low memory impact and fast computation. The metric measures mean distance between two pointclouds. However, we found that for our task it was not able to learn the features properly causing vertices to congregate together instead of spreading uniformly around the object shape. For this reason, we chose to use Earth Mover’s Distance (see Equation (7)) with expansion penalty (see Equation (8)), as per suggested penalization criteria for surface regularization proposed in MSN, where d(u,v) is Euclidean distance between two vertices in three-dimensional space and ϕ is the bijection of pointclouds. 𝟙 is the indicator function used to filter which shorter than $\lambda l_{i}$ with λ=1.5 as per suggested value, giving us a final combined reconstruction loss as final Equation (9) with α=0.1, ${\hat{S}}_{coarse}$ is coarse decoder output and ${\hat{S}}_{final}$ is final decoder output.

(7) $$\epsilon_{emd}\left(S,\hat{S}\right)=\underset{\phi :S\to \hat{S}}{min}\frac{1}{\left|S\right|}\sum_{x\in S}||x-{\phi \left(x\right)||}_{2}$$

(8) $$\epsilon_{exp}=\frac{1}{KN}\sum_{1\leq i\leq K}\sum_{\left(u,v\in \tau_{i}\right)}𝟙\{d\left(u,v\right)\geq \lambda l_{i}\}d\left(u,v\right)$$

(9) $$\epsilon =\epsilon_{clip}+𝟙\left\{\epsilon_{clip}<0.3\right\}\left(\epsilon_{emd}\left(S,{\hat{S}}_{final}\right)+\epsilon_{emd}\left(S,{\hat{S}}_{coarse}\right)+\alpha \epsilon_{exp}\right)$$

### 3.4. Dataset

**Figure 2:**
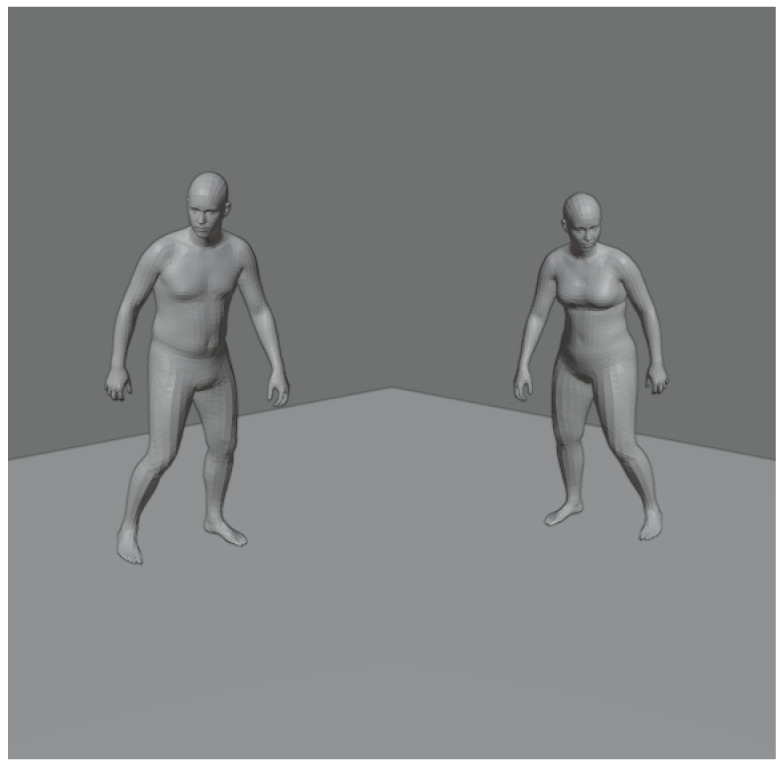
An example MoVi dataset motion capture pose applied to models provided by AMASS. The same pose is applied to female and male body type.

There are various existing datasets for object detection that contain labeled image data such as COCO [53] and Pascal VOC [54], 3D object datasets such as ShapeNet and even labeled voxel data [55]. However, our task required a very specific dataset: it required human meshes that could be used as ground truth, and it needed to contain depth camera information matching the mesh positions. As far as we are aware there exists no publicly available dataset matching this description. For this reason we generated a synthetic dataset using Blender [56]. The MoVi [57] dataset contains a vast amounts of motion capture data and multiple camera perspective video. However, videos contain no depth information, therefore it does not fully match our criteria. For this reason we used motion capture data bound to the AMASS [58] triangle meshes. An example of AMASS dataset can be seen in Figure 2.

**Figure 3:**
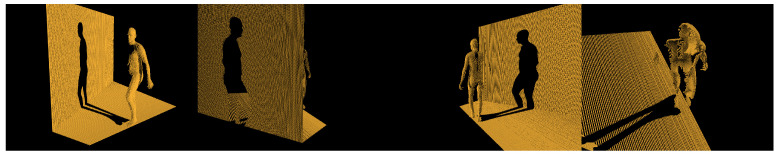
An example of neural network input that is created rendered depth frame converted to pointclouds with the help of camera intrinsic matrix *K*.

To create the dataset we placed the motion captured model into it and capture depth frames from various angles by rotating the camera and the person model itself. Rotating the camera simulated multiple cameras seeing same event, while rotating the model emulated the person doing same poses from different angles (see Figure 3). The person was rotated from 0° to 360° in the increments of 45°, while the camera was rotated from −35° to 35° in the increments of 15°. The camera was placed 4.5 m away from the person. The rendered depth frame was saved using OpenEXR [59] file format as unlike other general purpose image formats, such as JPEG, it is linear and lossless therefore it does not lose any depth information and is not limited to 8 bits per channel. Additionally, the frame itself was rendered using mesh and our ground-truth demands for pointcloud, to generate it we used uniform random sampling.

## 4. Results

### 4.1. Clipping Results

**Figure 4:**
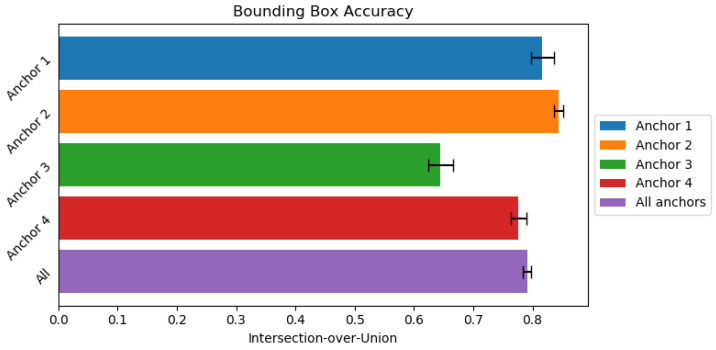
Bounding box accuracy expressed as I∩U for anchors. Higher is better.

In order to evaluate the accuracy of our clipping node we used Jaccards index [60,61,62] to compare the quality of our three-dimentional bounding boxes, which is widely adopted as a metric to compare bounding boxes. Our results (seen in Figure 4) indicate that for most of our anchors but one our I∩U≈80%, with overall accuracy being 79.07%, with some clipping error was able to be improved by slightly expanding the bounding boxes thus potentially improving bounding boxes which were very close to ground truth. The Jaccard index of Anchor 3 being much lower than others may be due to imbalanced number of samples belonging to each dataset.

### 4.2. Reconstruction Results

**Figure 5:**
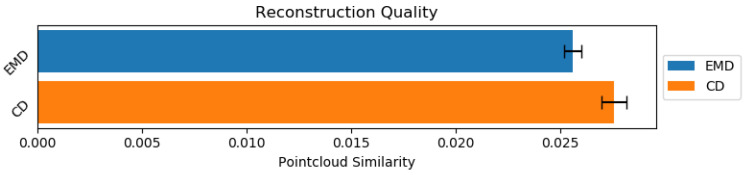
Reconstruction similarity using both Earth Movers Distance and Chamfer Distance. Lower value is better.

The purpose of our network was to reconstruct the human body shapes. To determine the quality of our reconstructions we needed an objective metric to compare results. For this reason we used two main metrics to evaluate model quality Chamfer Distance and Earth Movers Distance (Equation (7)), which is summarized in Figure 5.

(10) $$\epsilon_{cd}\left(S,\hat{S}\right)=\frac{1}{2}\left(\frac{1}{\left|S\right|}\sum_{x\in S}\underset{y\in \hat{S}}{min}||x-{y||}_{2}^{2}+\frac{1}{\hat{S}}\sum_{y\in \hat{S}}{\underset{x\in S}{min}||x-y||}_{2}^{2}\right)$$

**Figure 6:**
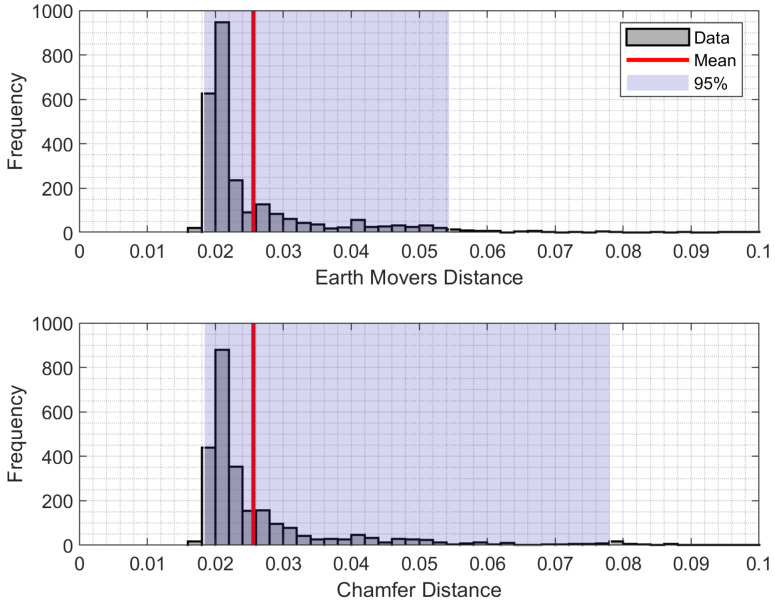
Distribution of Earth Movers Distance and Chamfer Distance values. Box shows 95% of values are between 2.5% and 97.5% percentiles of values.

We also summarize the distribution of the errors in terms of histogram as Figure 6. 95% of Earth Movers Distance was lower than 0.054, and for Chamfer Distance, lower than 0.078.

We cannot directly compare our results to other researchers’ reconstruction results, due to us using a completely different dataset than other state-of-the-art research uses. The approaches we have tested were unable to deal with the additional noise our dataset contains in the form of backgrounds and depth shadows as they lacked a Region of Interest mechanism. However, if we compare the metrics provided with other state-of-the-art methods (see Table 5) we can see that our reconstruction results were similar with the added robustness and flexibility by only reconstructing RoIs.

**Figure 7:**
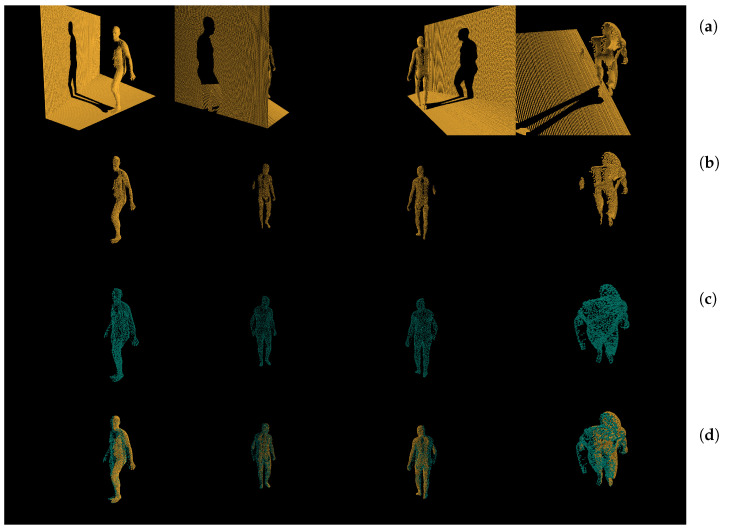
Different viewpoints of same pointclouds. Contains stacked from top to bottom: (**a**) input pointcloud; (**b**) clipped and sampled pointcloud; (**c**) predicted pointcloud; (**d**) combined (**b**,**c**).

**Figure 8:**
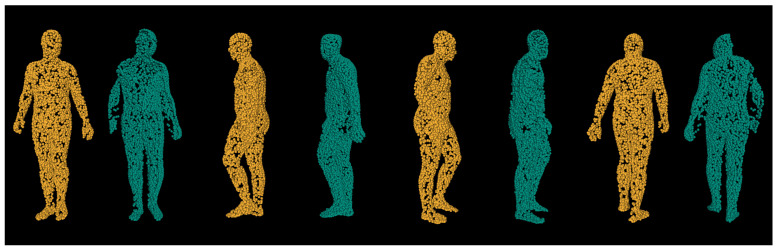
Comparison of ground truth (left/orange) and prediction (right/teal) from different viewpoints.

Another way to inspect prediction results that is not objective, nonetheless very important, is visually. Figure 7 displays same pointclouds from four different angles. The first row contains different views of input pointcloud that the first tier network responsible for clipping and resampling was fed. Once the prediction was made, the second row displays the pointcloud after clipping removed points that did not belong to the Region of Interest and downsampled them to 4096 points. The third row is the prediction made by the second tier network, responsible for the human body reconstruction. The final row shows first and second tier network results overlapped. As we can see, the prediction network managed to rebuild entirely missing features based on the most probable guess. Due to depth self-obstruction depth shadows were cast. This caused the input frame to be missing these features: half of the torso, half left hand, half of left hand, almost entire right hand, and half right leg. As we can see the prediction managed to guess very realistic right leg and right arm orientations based on the very few points that were provided by such features as the angle of the right shoulder and elbow. From this we can assert that our network had human-like speculative probabilities on how the obstructed parts of the body may be orientated. Additional validation of this assertion can be seen in Figure 8 comparing ground truth pointcloud and prediction made by the deep neural network. As we can see while there were imperfections in the predicted pointcloud the reconstructed object did in fact reconstruct the entire object shape.

**Figure 9:**
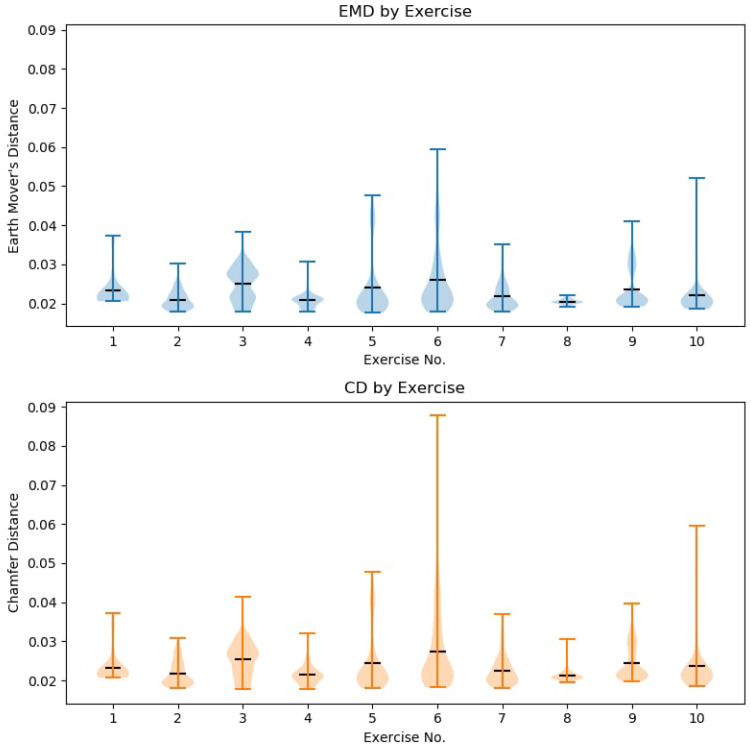
Reconstruction quality breakdown by the recorded motion capture exercise.

**Figure 10:**
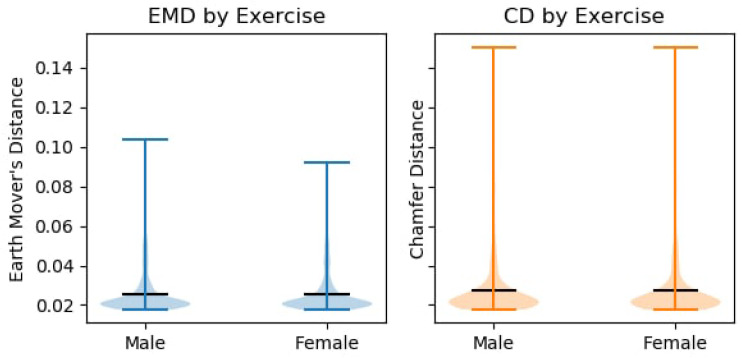
Reconstruction quality breakdown of the subjects’ by gender.

If we break down the reconstruction results by the pose, which is presented in Figure 9, we can see that the majority of our poses fell bellow 0.05 value of EMD and CD. Therefore the neural network was in fact able to perform pattern matching to the human pose. In further breakdown of our results (see Figure 10), we can see that there was very little disparity between the gender results, too. This implies that the suggested solution was body shape agnostic, as it was able to reconstruct both male and female human body shapes that were provided by the AMASS dataset with similar results. While a part of this gender reconstruction similarities can be attributed to the general similarities of the human shape, further visual inspection shows that the network was able to restore the distinctly male or female features.

## 5. Discussion and Concluding Remarks

### 5.1. Discussion

The main advantage of the proposed two-tiered neural network architecture as compared to existing reconstruction algorithms is the addition of the first tier Region of Interest (RoI) extraction node. Existing object reconstruction implementations deal with pre-masked user data. Therefore, they are not fit for real-world-like input data, where additional background noise exists along with the object we are attempting to reconstruct. Unfortunately, in addition to background noise, real-world depth sensors also produce a lot of distortions in their depth frames, for which our approach was not able to account for. This requires further research in the field by either creating a real world dataset akin to our synthetic, or an attempt to recreate the distortions for the synthetic dataset which could be used as an augmentation. Additionally, our RoI node is not strongly coupled to the reconstruction branch. This allows us to replace one part of the model completely without retraining the other. For example, our current implementation is unable to extract multiple Regions of Interest from a depth frame. However, if such changes were to be applied, we would be able to keep the existing reconstruction weights. This would allow us to run a separate reconstruction task for each region of interest, without changing the entire reconstruction network architecture, thus reducing the amount of Graphical Processing Unit (GPU) time required to train it. Finally, unlike a lot of previous methods, that attempt to rebuild the object shape using voxel grid, non-normalized pointcloud approach inherently does not need to solve for homography, which removes the requirement of extracting the objects world transformation matrix. Instead, the pointcloud based approaches that do not apply normalization to the pointcloud in attempt to improve training process, reconstruct the vertices in their positions in relation to camera space. This removes the need of translating world space coordinates into camera space post-reconstruction and therefore can be easily applied in such applications as Virtual Reality in conjunction with Augmented Reality.

### 5.2. Concluding Remarks

We have proposed a two-tiered neural network architecture which has successfully achieved the desired goal of reconstructing human shaped pointcloud.

The proposed network achieved Jaccards’ Index of 0.7907 for the first tier which is used to extract Region of Interest from the pointcloud. Second tier of the network has achieved Earth Movers distance of 0.0256 and Chamfer distance of 0.276 indicating good experimental results. Further, subjective reconstruction results inspection shows strong predictive capabilities of the network, with the solution being able to reconstruct limb positions from very few object details.

Finally, unlike previous research, due to the use of anchor boxes our solution does not rely on the user given mask in order to perform reconstruction step giving us a clear advantage over other approaches and theoretical ability to reconstruct multiple objects per scene.

### 5.3. Future Work

Our current implementation has been trained and tested using a noiseless synthetic dataset only. Real world depth frames generally contain a lot of imperfections when using consumer grade sensors for that reason future work would have to adapt the proposed solution to be able to reconstruct real world data. Producing such a dataset is a tedious task as it requires labeling 3D data by manually extracting the three-dimensional bounding boxes from a given depth frame in addition to creating an appropriate pointcloud representations to be used as ground truths during the training process. The later can be achieved by creating a dataset containing pointcloud fusion of multiple camera perspectives. Additionally, our dataset only deals with the reconstruction of a single object, where there are no additional objects in the scene, therefore a human body which is occluded by other objects within the scene would not be properly reconstructed.

## Figures and Tables

**Table 1:** Table comparing different existing implementations. Standalone refers to sensor-to-screen solutions where for any given sensor input a fully reconstructed model can be expected without inputting external information that the sensor itself cannot provide, such as masks.

Name	Voxels	Pointcloud	Input	EMD	CD	Standalone
3D-R2N2	✓	✗	RGB	—	—	✗
YoloExt	✓	✗	RGB-D	—	—	✓
PointOutNet	✗	✓	RGB	✓	✓	✗
PointNet w/FCAE	✗	✓	Pointcloud	✗	✓	✗
PCN	✗	✓	Pointcloud	✗	✓	✗
AtlasNet	✗	✓	Pointcloud	✗	✓	✗
MSN	✗	✓	Pointcloud	✓	✗	✗
Ours	✗	✓	Depth	✓	✗	✓

**Table 2:** Architecture of the clipping network. The last convolutional layer does not contain the activation function because finding bounding boxes is a regression task.

Type	Filters	Size	Output
Depth	-	-	640×480
Pointcloud	-	-	307,200×3
Resample	-	-	2048×3
Convolution 1D	64	1	2048×64
Convolution 1D	128	1	2048×128
Convolution 1D	1024	1	2048×1024
Adaptive Max Pool 1D	-	2	2×1024
Convolution 1D	512	1	2×512
Linear Convolution 1D	7	1	2×7
Clip Inputs	-	-	307,200 × 3
Resample	-	-	4096×3

**Table 3:** Architecture of the reconstruction neural network. We use 16 Morph-Based-Decoders for 16 potential surfaces for the network to be able to predict.

Label	Type	Filters	Size	Output
	Input	-	-	4096×3
Encoder	Convolution 1D	64	1	4096×64
	Convolution 1D	128	1	4096×128
	Linear Convolution 1D	1024	1	4096×1024
	Max Pool 1D	-	-	1024
	Fully Connected	256	-	256
16 × Coarse Decoder	Convolutional 1D	256	1	16×256×258
	Convolutional 1D	129	1	16×256×129
	Convolutional 1D	64	1	16×256×64
	Convolutional 1D	3	1	16×256×3
	Concatenation	-	-	4096×3
Final Decoder	Convolutional 1D	64	1	4096×64
	Convolutional 1D	128	1	4096×128
	Convolutional 1D	1024	1	4096×1024
	Max Pool 1D	-	-	1024
	Residual	-	-	1088
	Convolutional 1D	512	1	4096×512
	Convolutional 1D	256	1	4096×256
	Convolutional 1D	128	1	4096×128
	Convolutional 1D	3	1	4096×3

**Table 4:** Comparison of neural network complexity by number of parameters, number of operations and model size.

Method	No. of Parameters (M)	No. of Operations (GFLOPs)	Model Size (MB)
PointNet w/FCAE	7.43	1.18	28.36
PCN	6.87	29.5	26.25
AtlasNet	3.31	6.46	12.66
MSN	29.50	12.89	112.89
Ours	29.71	11.74	112.94

**Table 5:** Reconstruction metric comparison between other methods and ours. While direct comparison cannot be drawn due different datasets and techniques being adopted we can see that the reconstruction values are at least very similar to state-of-the-art when compared to ShapeNet dataset. As per Liu et al. (2020) reference values.

Method	EMD	CD	Dataset
PointNet w/FCAE	0.0832	0.0182	ShapeNet
PCN	0.0734	0.0121	ShapeNet
AtlasNet	0.0653	0.0182	ShapeNet
MSN	0.0378	0.0114	ShapeNet
Ours	0.0256	0.0276	AMASS

## Data Availability

Data is available from the corresponding author upon reasonable request.
